# Regional anaesthesia in the emergency centre: Knowledge, attitudes and practices of doctors in the Western Cape, South Africa

**DOI:** 10.4102/safp.v67i1.6194

**Published:** 2025-11-14

**Authors:** Andrew D. Fourie, Adéle de Goede, Michael Pather

**Affiliations:** 1 Department of Family and Emergency Medicine, Faculty of Medicine and Health Sciences, Stellenbosch University, Cape Town, South Africa; 2 Department of Anaesthesiology and Critical Care, Faculty of Medicine and Health Sciences, Stellenbosch University, Cape Town, South Africa

**Keywords:** regional anaesthesia, peripheral nerve blocks, emergency centre, multi-modal analgesia, knowledge, attitudes, practices, training, South Africa

## Abstract

**Background:**

Regional anaesthesia in emergency centres (ECs) offers clear benefits for patient care and system efficiency. However, its use in South African ECs remains inconsistent, and the knowledge, attitudes and practices of emergency doctors are not well understood. A notable gap exists in formal training.

**Methods:**

A cross-sectional survey was conducted among EC doctors at four district hospitals in the Metro-East health district of the Western Cape, South Africa. Data were collected via an electronic questionnaire exploring knowledge, attitudes and practices related to regional anaesthesia.

**Results:**

Eighty-five doctors participated (58% response rate). Among them, 67.1% had prior training in peripheral nerve blocks, predominantly informal and peer-based (98.2%), with only 8.8% receiving formal training. Most (96.5%) expressed interest in further training. Regional anaesthesia was underused: 36.5% used it less than monthly, 23.5% weekly and only 2.4% daily. Despite this, 77.6% rated it as important or very important, and 75.3% scored its usefulness ≥ 7/10. Barriers included limited confidence in managing local anaesthetic toxicity and inconsistent monitoring: 39.1% never monitored, while only 25% did so consistently. The most cited barrier (92.9%) was a lack of knowledge.

**Conclusion:**

Despite strong interest and favourable attitudes, regional anaesthesia remains underutilised because of limited knowledge, informal training and concerns about safety and monitoring.

**Contribution:**

This study reveals a disconnect between positive clinician attitudes and limited practical competence. It underscores the need for structured, formal educational initiatives to support safe and effective regional anaesthesia use in district ECs.

## Introduction

Pain management is a fundamental aspect of emergency medicine, as pain ranks among the most common presenting complaints in the emergency centre (EC).^1,2^ Despite this, evidence suggests that pain is often inadequately managed, with suboptimal analgesia frequently provided.^1,2,3^ The importance of adequate pain control extends beyond immediate patient comfort, as poorly managed acute pain is associated with increased morbidity, prolonged EC stays and greater healthcare costs.^2,4,5^ Consequently, research into optimal pain management strategies has intensified, particularly focusing on multimodal analgesia, opioid-sparing techniques and regional anaesthesia.^1,6,7,8,9^

The concept of regional anaesthesia is the umbrella term applied to both neuraxial anaesthesia and peripheral nerve blocks.^10,11,12^ In both neuraxial and peripheral techniques, infiltration of the nerve or plexus with local anaesthesia results in a block of the nerve impulse transmission, thereby preventing or relieving pain.^1,13^ The benefits of regional anaesthesia are well established in the perioperative period and provide numerous advantages for the patient, yet these benefits extend beyond the operating theatre and are increasingly being recognised for their role in the EC setting.^10,11,12,14,15^

Which of the myriad options (and ever-expanding smorgasbord) of peripheral nerve blocks to use remains an open question. The concept of ‘plan A blocks’ is described in the anaesthetic literature^16^ and, with minor adjustments, can be applied to the EC setting, even in austere environments.^17^ The provision of peripheral nerve blocks in the EC has been shown to improve pain control, reduce opioid requirements, reduce hospital length of stay, save costs and enhance patient satisfaction.^18,19,20,21,22,23^ However, the safe and effective use of these techniques requires specific knowledge of anatomy, local anaesthesia agents, ultrasound utilisation and procedural skills – areas where emergency doctors often receive limited formal training.^17^

The innovation of ultrasound-guided regional anaesthesia (UGRA) revolutionised the field of regional anaesthesia.^24^ It has gained widespread acceptance in both the anaesthesia and emergency medicine communities, along with substantial evidence supporting its efficacy.^10,11,12,14,25,26^ Research has consistently shown that UGRA reduces complications, shortens block onset time and improves patient outcomes compared to traditional landmark-based techniques.^27,28^ Systematic reviews and meta-analyses have since reinforced the value of peripheral nerve blocks in emergency medicine, demonstrating efficacy in reducing opioid consumption, decreasing hospital length of stay and improving patient satisfaction.^6,29,30,31^

In high-income countries, UGRA has increasingly been adopted in emergency care.^22,23,29,30,31,32,33^ However, international studies indicate that its uptake remains inconsistent and is often underutilised despite favourable outcomes.^18,34,35^ In low- and middle-income countries (LMICs), such as South Africa, similar trends have been observed.^36,37,38,39,40,41,42^ Factors contributing to limited usage include insufficient training, lack of access to ultrasound equipment and the absence of institutional protocols or evidence-based clinical guidelines.^1,36,37,39,40,41,42^

International evidence suggests that even brief, targeted training sessions can improve doctors’ competence in regional anaesthesia.^7^ Positive clinician attitudes towards regional anaesthesia have been documented in Africa. However, significant knowledge and skills gaps persist.^36,38,39,43^ The absence of South African EC-specific guidelines and training initiatives further complicates the issue. While organisations such as the South African Society of Regional Anaesthesia have produced comprehensive perioperative guidelines,^44^ these do not adequately address the unique requirements of the EC environment. Similarly, the Emergency Medicine Society of South Africa offers practice guidelines for procedural sedation in the EC, but not for regional anaesthesia.^45^

Although the benefits of regional anaesthesia are well established, limited evidence exists regarding its practical implementation by non-anaesthetists, as most existing research focuses on anaesthesiology trainees.^15,29,38,39,40,41,44,46,47^ Emergency centre doctors’ training, experiences and needs, particularly in LMICs like South Africa, remain largely unexplored. Moreover, the literature provides limited insight into the real-world use of peripheral nerve blocks by EC doctors, including their confidence, perceived barriers and clinical practices.^48,49^

In the South African context, district hospital ECs are often staffed by generalist doctors who have varying levels of training in the procedural skills associated with regional anaesthesia. The lack of local data represents a critical knowledge gap, especially given the increasing availability of ultrasound equipment and the focus on improving pain management in emergency care. Understanding how emergency doctors in South African district hospitals engage with regional anaesthesia, specifically UGRA, is essential to inform training programmes, institutional policies and clinical guidelines to expand the safe use of these techniques.

This study aimed to evaluate the knowledge, attitudes and practices of doctors working in four district hospitals within the Metro-East Health District of Cape Town, South Africa, regarding the use of regional anaesthesia for analgesia in the EC. The primary objectives were to assess doctors’ knowledge of the types of peripheral nerve blocks commonly used in the emergency setting, to explore attitudes towards their use and to describe their current practices in this regard. Secondary objectives included evaluating the nature of doctors’ training in peripheral nerve blocks, their level of satisfaction with this training and identifying systemic or other barriers to implementing regional anaesthetic techniques in the EC.

## Research methods and design

### Study design

This study employed an observational, cross-sectional design with descriptive and comparative components.

### Setting

The research was conducted at the four district hospitals within the Metro-East health district of Cape Town, South Africa: Eerste River Hospital (ERH), Helderberg Hospital (HH), Khayelitsha District Hospital (KDH) and Karl Bremer Hospital (KBH). All four district hospitals within the district were included in the study. The district hospital serves as the primary referral point for healthcare facilities in the district, forming an integral link in the healthcare system between primary healthcare and secondary and tertiary services, and is responsible for the bulk of the healthcare services rendered to patients. Each is staffed by a varying number of intern doctors, community service medical officers, medical officers, registrars and consultants. They are heterogenous in their staffing components working in their respective ECs: for example, ERH and HH have large generalist populations, and almost all doctors working in these hospitals perform some of their clinical duties in the EC. In contrast, KDH and KBH have dedicated (and therefore smaller) staffing complements working in the EC with far more departmental separation in other clinical domains.

### Sample size and selection

The study population comprised all doctors with clinical responsibilities in the EC of the four district hospitals in the Metro-East health district. This included intern doctors, community service doctors, medical officers, registrars, consultant family physicians and consultant emergency physicians. All levels of staff were included to investigate group differences between various clinical levels. Inclusion criteria were doctors holding an MBChB degree or higher who were actively involved in clinical duties within the EC. Doctors without any clinical responsibilities in the EC were excluded. A census sampling strategy was employed, with all eligible doctors invited to participate in the study. The estimated number of eligible participants across the four hospitals was approximately 147, distributed as follows: ERH, 45; HH, 60; KDH, 30; and KBH, 12. A target response rate of 60% (approximately 88 participants) was considered sufficient to address the study objectives and is comparable to similar survey-based studies.^50,51^

### Data collection

Data were collected using a researcher-adapted, structured questionnaire that collated elements largely from a fully validated survey,^52^ as well as from other knowledge, attitude and practice-type survey tools.^36,53^ The instrument comprised multiple-choice and Likert scale-type questions designed to explore several domains: knowledge of peripheral nerve blocks and local anaesthetic agents; attitudes towards the use of regional anaesthesia in the ED; self-reported practices, including frequency of use and procedural confidence; training history and satisfaction with training; and perceived barriers to implementation. To ensure content validity, a panel of six clinical experts reviewed and validated the questionnaire, consisting of three consultant family physicians and three consultant anaesthesiologists. A pilot study was conducted with 10 doctors to evaluate for clarity and consistency. Eligible participants were identified through lists obtained from clinical managers at each participating hospital. The survey was distributed electronically via WhatsApp, accompanied by a link to the RedCap platform, and included an information sheet and an informed consent form. Reminder messages were sent, where individual contact details were available, to encourage participation.

### Data analysis

Data were exported from RedCap and analysed using IBM® SPSS® Statistics Version 30.0. Depending on the data distribution, descriptive statistics included frequencies, percentages, means (standard deviation) or medians (interquartile range).

### Ethical considerations

Ethics approval was obtained from the Health Research Ethics Committee (HREC) at Stellenbosch University (Reference number: S24/07/171), and permission to conduct the research was obtained from the Department of Health, Government of the Western Cape (Reference number: WC_202409_044). Written informed consent was obtained from the study participants. Data were securely maintained on the RedCap electronic platform. Any downloaded data were deidentified and stored on a secure cloud server affiliated with the Department of Family and Emergency Medicine at Stellenbosch University.

## Results

### Participant demographics

**FIGURE 1:**
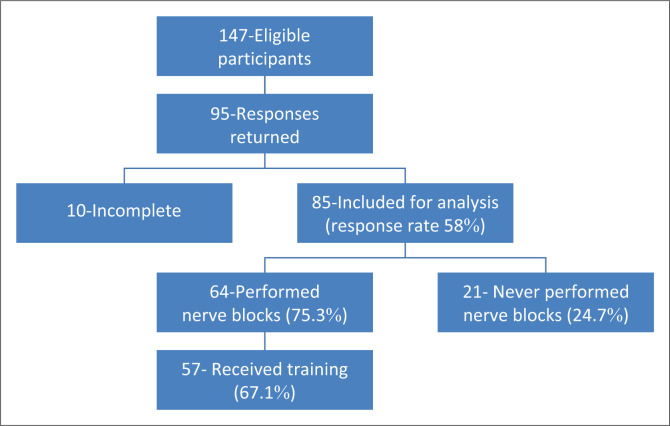
Eligible participants, performance of nerve blocks and training received.

Of the 147 eligible emergency doctors, 95 responses were returned, of which 10 were incomplete and excluded, with 85 included in the final analysis (Figure 1). The cohort had a median professional experience of 5.5 years (1–30), reflecting an early-to-mid-career workforce, with a representative sampling of various professional designations. Most participants worked at HH (64.7%), with smaller representation from ERH (20.0%), KBH (8.2%) and KDH (7.1%). Only 9% (8) of the participants held specialisation degrees (six family physicians and two emergency physicians), underscoring the generalist nature of the workforce (Table 1).

**TABLE 1:** Demographic and professional characteristics of participants.

Characteristic	*N* = 85		
	Median	*n*	%
**Age (years)**			
25–54	29	-	-
**Years of experience**			
1–30	5.5	-	-
**Specialisation degree**	-	8	9.0
**Professional designation**			
Interns	-	25	29.4
Community service officers	-	18	21.2
Medical officers	-	23	27.1
Registrars	-	13	15.3
Consultants	-	6	7.1
**Affiliated hospital**			
Helderberg Hospital	-	55	64.7
Eerste River Hospital	-	17	20.0
Karl Bremer Hospital	-	7	8.2
Khayelitsha District Hospital	-	6	7.1

### Training and competency in regional anaesthesia

Of the 85 participants surveyed, 67.1% reported having received prior training in regional anaesthesia (Figure 1). Of those, the most frequently reported training modality was senior or mentor-led instruction (98.2%). In contrast, relatively few participants reported exposure to offline materials (31.6%), digital resources (24.6%), or formal courses (8.8%). Satisfaction with this training was suboptimal: 47.4% felt neutral, 31.6% were satisfied and only 5.3% were very satisfied with the training received.

**FIGURE 2:**
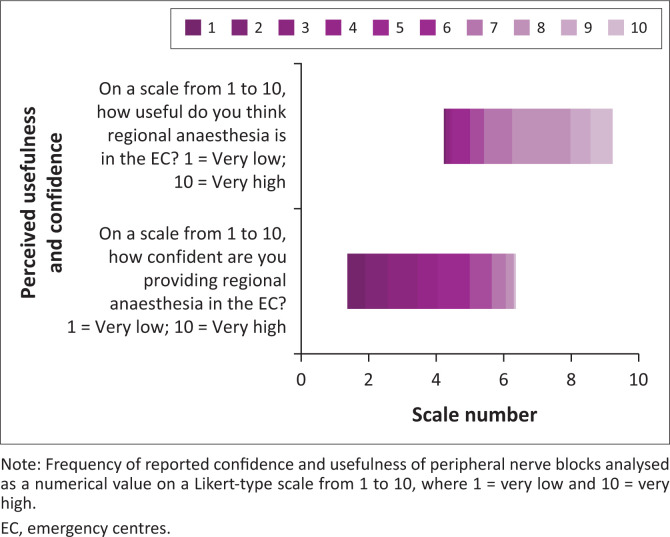
Perceived usefulness vs confidence of peripheral nerve blocks in the emergency centres.

Overall, a clear mismatch was evident between participants’ perceived confidence (median 4.0) and perceived usefulness (median 8.0) of regional anaesthesia, as noted in Figure 2.

**FIGURE 3:**
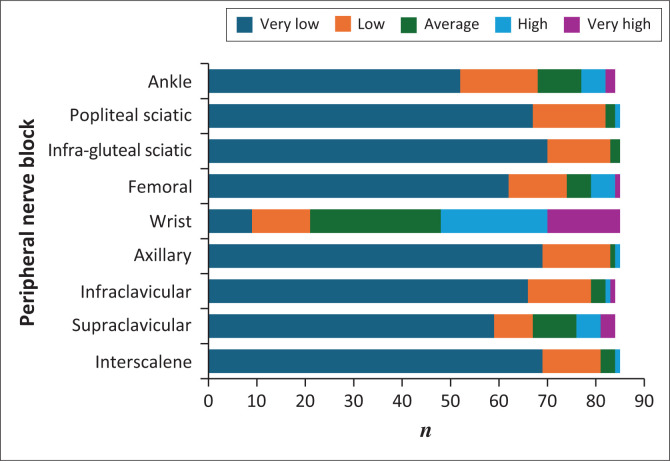
Confidence with peripheral nerve blocks.

**FIGURE 4:**
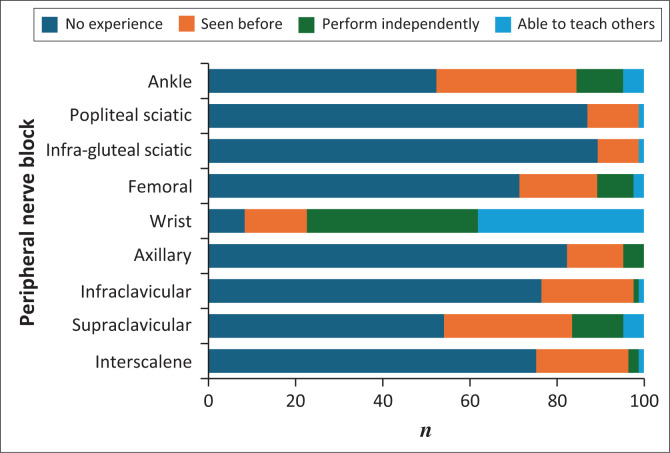
Experience with peripheral nerve blocks.

While the wrist block emerged as a notable exception, confidence and experience with specific peripheral nerve blocks were low. As noted in Figure 3, 75.3% reported at least average (31.8%), high (25.9%), or very high (17.6%) confidence in performing the wrist block. Furthermore, 76.5% of participants indicated that they could either perform the wrist block independently (38.8%) or teach others (37.6%) (Figure 4). In contrast, confidence and experience levels were significantly lower for all other blocks. The interscalene brachial plexus block, for example, was reported to have very low confidence by 81.2% of participants, and no experience was reported by 75.3%. Similar patterns were seen with all other peripheral nerve blocks (Figure 3 and Figure 4).

### Attitudes

Despite these limitations, 96.5% of participants reported that teaching and training would encourage them to incorporate regional anaesthesia into their EC practice. A total of 88.3% of participants viewed such training as either important (45.9%) or very important (42.4%). Furthermore, a majority of participants (77.6%) believed that peripheral nerve blocks were either important (48.2%) or very important (29.4%) for their work in the EC.

Participants reported a strong interest in expanding the use of peripheral nerve blocks, with 50.8% expressing a desire to add the infra-gluteal sciatic block, 50.8% the axillary brachial plexus block, 56.9% the popliteal sciatic block, 58.5% the femoral nerve block, 61.5% the supraclavicular brachial plexus block and 70.8% the ankle block to their EC practice.

### Clinical utilisation and perceived barriers

In terms of clinical utilisation, regional anaesthesia appeared underutilised in ECs. Thirty-six per cent of participants administered peripheral nerve blocks less than once per month, 23.5% administered blocks weekly and only 2.4% used them daily. Motivators for use included improved pain relief (89.4%) and patient safety (65.9%).

The most frequently cited perceived disadvantages of peripheral nerve blocks in the EC were the time required to perform or establish a block (62.4%) and the pain or tolerability of the procedure (48.2%). Fewer participants cited concerns about nerve injuries (35.3%), failed blocks (29.4%), or local anaesthetic toxicity (29.4%). When asked about their preferred analgesia option, 41.2% of participants favoured procedural sedation, 23.5% peripheral nerve blocks, 25.9% a combination of the two and 9.4% would follow patient preference.

The main perceived barrier to peripheral nerve block use in the EC was a reported lack of knowledge and experience, cited by 92.9% of participants. Other operational barriers included time constraints (70.6%), equipment issues (32.9%), stock shortages (30.6%), patient compliance (21.2%) and cost considerations (5.9%). Notably, 100% of respondents reported having access to an ultrasound machine in their EC.

### Practices and knowledge gaps

Regarding the management of local anaesthetic systemic toxicity, only 37.6% of participants reported that the rescue therapy, lipid emulsion, was stocked in their EC, 14.1% reported it was not and 48.2% were unsure. Of those who confirmed availability, 43.8% knew of its location. Additionally, 74.1% of participants reported either low confidence (60.0%) or being unsure (14.1%) of the management of local anaesthesia systemic toxicity.

A total of 75.3% of participants had previously performed a peripheral nerve block, while 24.7% had not (Figure 1). Among those who had, verbal consent was most frequently obtained in 73.4% of cases. A majority (67.2%) of participants reported not performing blocks on children. Nearly half (47.6%) of patients were never sedated prior to undergoing a block. Worryingly, 39.1% reported never monitoring patients post-block, compared to 25.0% who reported consistent monitoring, 1.6% for paediatric patients only, 20.3% for high-risk patients only, and 14.1% for both paediatric and high-risk patients. Monitoring practices varied: 53.1% of participants monitored patients for 15 min or more, 35.9% for at least 30 min and only 10.9% for longer than 30 min.

Among the 75.3% of participants who had performed peripheral nerve blocks (Figure 1), aseptic technique practices also varied. Adhesive films were the most reported method of probe asepsis (53.1%), followed by sterile gloves (28.1%). Few used specially designed probe covers (6.3%), and 7.8% reported using no probe asepsis at all. Further aseptic procedures included skin preparation (89.1%) and the use of sterile gloves (87.5%), with a wide range of reported use. In total, 71.4% of participants reported no limitations in their aseptic practice. Most respondents (77.8%) report not using an assistant to inject local anaesthetic for them. Documentation typically occurred in EC notes (93.7%), with minimal use of anaesthetic charts (4.8%).

Few participants had witnessed complications resulting from peripheral nerve blocks. Reported adverse events included nerve injury (3.2%), local anaesthetic toxicity (4.8%), wrong-side blocks (6.3%), pneumothorax (7.9%) and bleeding (22.2%). Most (61.9%) had never observed a complication during their EC practice. When asked if witnessing a complication led to a change in practice, a majority (61.9%) reported no change in practice, while 33.3% reported an effort to improve knowledge or skills.

## Discussion

This study provides valuable insights into the current state of regional anaesthesia practice among emergency doctors in Western Cape district hospitals. It highlights significant gaps in training, confidence and clinical utilisation. The findings underscore the need for structured educational interventions and systemic support to enhance regional anaesthesia adoption in ECs.

The results reveal a paradox: while most participants reported prior training in regional anaesthesia, satisfaction levels were low, along with confidence and experience with all peripheral nerve blocks, except for the wrist block, a finding that echoes international data.^54^ This paradox suggests that current training methods may not foster procedural competence. The finding of near-exclusive reliance on informal collegial mentorship, with minimal use of formal courses or digital resources, stands at odds with other published research,^35,52^ and indicates an urgent need for more standardised, formal and accessible training modalities. Simulation-based training, structured workshops and online modules could supplement traditional mentorship, thereby improving confidence and skill retention.

The high proportion of participants expressing a desire for further training and recognising the importance of regional anaesthesia in the EC reinforces the potential for educational interventions to bridge this gap. An overwhelming majority of participants believe that training in regional anaesthesia was important, with a striking mismatch evident between participants’ perceived level of confidence and the perceived usefulness of regional anaesthesia. These findings highlight positive attitudes towards an underutilised practice. Because most participants were early-to-mid-career professionals, integrating peripheral nerve block training into postgraduate curricula and continuing professional development programmes could yield long-term benefits.

Despite its proven benefits for pain management in the EC, this study demonstrates that regional anaesthesia remains underutilised in ECs, with only a minority of practitioners administering peripheral nerve blocks weekly or daily, consistent with published research.^18,34,35^ The primary barriers, including lack of knowledge, limited experience and time constraints, suggest that logistical and educational factors, rather than scepticism about efficacy, limit adoption. Ultrasound access was not a limiting factor in this cohort, with universal access reported.

Interestingly, concerns about complications (e.g. nerve injuries and local anaesthetic toxicity) were less prominent than expected, with procedural time and patient tolerability being greater deterrents. This contrasts with literature emphasising safety concerns as a significant barrier,^35^ suggesting that practical and operational challenges in this cohort outweigh theoretical risks. Addressing these barriers requires not only training but also systemic changes, such as streamlining block performance processes, ensuring equipment availability and optimising workflow to accommodate regional anaesthesia procedures.^54^

Several critical safety gaps were identified, including inconsistent monitoring practices, uncertainty about lipid emulsion availability and low confidence in managing local anaesthetic systemic toxicity. These findings align with previous studies highlighting variable adherence to regional anaesthesia safety protocols in resource-limited settings.^39,40,42,53^ A significant proportion of participants reported never monitoring patients post-block, which is particularly concerning and warrants urgent attention. Documentation practices were also inadequate, with most practitioners relying on EC notes instead of standardised anaesthetic charts. Improved documentation protocols, along with mandatory monitoring guidelines, could enhance patient safety and facilitate audit processes.

While most participants adhered to basic aseptic techniques (skin preparation, sterile gloves), the infrequent use of dedicated probe covers suggests room for improvement in infection control, while acknowledging the resource constraints within the South African healthcare system. The low reported complication rates may reflect underutilisation rather than true safety, as complications are more likely with increased procedural volume. Nevertheless, the expressed interest in expanding peripheral nerve block use (e.g. ankle, femoral and sciatic blocks) indicates a willingness to adopt regional anaesthesia if supported by adequate training and resources.

## Limitations

This study has limitations that should be considered when interpreting the findings. Firstly, the sample size (*n* = 85) was relatively small and restricted to four district hospitals in Cape Town’s Metro-East health district, South Africa, which may limit generalisability to other regions or secondary and tertiary care settings. Secondly, three of the four district hospitals included in the study had poor response rates, which may impact the results. Thirdly, the target response rate of 60% was not achieved. Fourthly, self-reported data on competency, practices and complications are susceptible to recall and response bias, as participants may overestimate their confidence or underreport adverse events. Lastly, the cross-sectional design prevents causal inferences about barriers to regional anaesthesia adoption. Future multi-centre studies with longitudinal designs and mixed-method approaches (e.g. procedural audits, patient interviews) could address these gaps.

## Recommendations

To address the critical gaps in knowledge and skills, structured training programmes in regional anaesthesia should be implemented for all EC doctors working in district hospitals. These programmes should combine theoretical instruction with hands-on, supervised practice, ideally developed in collaboration with anaesthesiology departments or regional anaesthesia specialists.^55^ Incorporating regional anaesthesia into continuous professional development curricula will support the maintenance of competency and ensure clinicians remain updated with evolving best practices.

Developing and adopting evidence-based, EC-specific protocols are essential for standardising practice. These guidelines should clearly define indications, contraindications, procedural steps and safety measures while considering local resource availability. Recently published literature can serve as a framework for tailoring a protocol to a specific hospital or setting.^17^ To support widespread adoption, protocols should be embedded into clinical workflows using accessible formats such as laminated quick-reference guides or digital tools integrated into electronic health systems.

Establishing a mentorship programme that pairs less experienced clinicians with skilled practitioners can help build confidence through supervised practice and immediate feedback. This should be supported by ongoing peer-learning opportunities, including simulation-based workshops and case discussions, reinforcing knowledge and fostering a collaborative learning culture.

Ensuring that ECs are equipped with the necessary tools, such as specialised needles and a consistent supply of local anaesthesia agents, is essential for safe and effective practice. Training initiatives focused on UGRA techniques will be critical, especially in resource-constrained environments.

Regional anaesthesia should be integrated into existing clinical governance structures, including morbidity and mortality reviews and routine clinical audits, to ensure effective implementation and optimal outcomes. Monitoring usage patterns, complications and implementation barriers through quality improvement initiatives can yield valuable insights to refine existing protocols and drive system-level improvements.

Future research should explore the long-term impact of standardised training interventions on clinician competency and patient outcomes. Comparative studies across different healthcare tiers (primary, regional and tertiary) could also identify systemic barriers and opportunities for scaling access to regional anaesthesia. By bridging these gaps, healthcare systems can harness the full potential of regional anaesthesia, improving the quality of emergency care and equitable pain management for underserved populations.

## Conclusion

This study highlights the knowledge, attitudes and practices of emergency doctors in Cape Town’s Metro-East district hospitals regarding regional anaesthesia. While clinicians expressed strong interest, positive attitudes and recognition of its clinical value, formal training was limited, with most knowledge being acquired through informal, collegial channels. Practices varied widely, particularly concerning block selection, aseptic technique, ultrasound asepsis, patient monitoring and preparedness for local anaesthetic toxicity. Such variability raises concerns about patient safety and suggests missed opportunities to optimise pain management and resource efficiency in emergency care.

Despite low utilisation rates, nearly all participants supported the inclusion of regional anaesthesia in EC care, citing perceived benefits such as improved pain control and patient safety, which highlight positive attitudes. Key barriers included inadequate training, time constraints and issues with equipment and stock availability. The strong interest expressed in regional anaesthesia signals a readiness for change among Western Cape EC healthcare professionals. This study serves as a call to action for policymakers, educators and clinicians to collaborate in making regional anaesthesia a viable, routine option for South African EC doctors.
